# Editorial for the Special Issue on Sensors and Wearable Technologies in Sports Biomechanics

**DOI:** 10.3390/s24196219

**Published:** 2024-09-26

**Authors:** Yih-Kuen Jan, Chi-Wen Lung, Ben-Yi Liau, Manuel E. Hernandez

**Affiliations:** 1 Department of Health and Kinesiology, University of Illinois at Urbana-Champaign, Urbana, IL 61801, USA; 2 Department of Creative Product Design, Asia University, Taichung 413305, Taiwan; cwlung@asia.edu.tw; 3 Department of Automatic Control Engineering, Feng Chia University, Taichung 407102, Taiwan; byliau@fcu.edu.tw; 4 Department of Biomedical and Translational Sciences, University of Illinois at Urbana-Champaign, Urbana, IL 61801, USA; mhernand@illinois.edu

Sport biomechanics is a subfield of biomechanics that studies mechanics to improve performance and reduce injury during exercise and sports competitions in abled-bodied people and people with disabilities. Traditionally, sport biomechanics research has been conducted in laboratory settings that limit the generalization of research findings to real-life field competitions. Recent advancements on sensors and wearable technologies have provided new opportunities to examine on-the-field performance and provide real-time, continuous feedback for coaches and athletes with and without disabilities [1,2]. These sensors and wearable technologies include physiological (e.g., heart rate and temperature), neurological (e.g., brain waves), biochemical (e.g., metabolites and biomarkers), and biomechanical (e.g., force and pressure) sensors. Although these sensors and wearable technologies have demonstrated the promise to improve sports performance and reduce injury in exercise and sports, these sensors have not been widely applied to study various sport activities (e.g., speed skating, football, and marathons) [3]. Many novel wearable sensors for assessing cardiovascular and metabolic processes have not been adopted to benefit athletes. Furthermore, data collected from these wearable sensors have not been fully analyzed using advanced machine learning algorithms. The objective of this Special Issue is to highlight novel sensors and wearable technologies and their applications in exercise and sports competitions, as well as the use of advanced machine learning (ML) and generative artificial intelligence (AI) for interpreting the collected continuous sensor data.

This Special Issue has contributed to addressing the gaps and challenges by documenting the application of wearable sensors on assessing performance and injury risk of athletes during various sports activities and the development and testing of novel wearable sensors. A total of 16 articles are included in this Special Issue including the development and validation of movement assessment devices (6 articles), wearable plantar pressure sensors (3 articles), movement classification (2 articles), water sports (2 articles), golf (1 article), snow skiing (1 article), and wheelchair sports (1 article). These studies have advanced the technique of developing sensors and wearable technologies for various sports. The use of wearable insole sensors provides a new and convenient way to assess the interactions of ground reaction forces and the foot. Several researchers also explored applying wearable sensors in various sports to better understand the performance of athletes as well as the risk for sports injury. In these included studies, sensors and wearable technologies have been demonstrated to benefit athletes by enhancing performance and minimizing injury risk. A missing link from these studies is the education of targeted users. Research studies usually reported the acquisition of these real-time, continuous physiological and biomechanical data without sufficient elaborations on how to use these data. The performance of athletes depends on the interactions between coaches and athletes [4]. There is a need to educate athletes and coaches to understand the meanings of these sensor data. Aligned with this topic, there is a need for further development and integration of generative AI and ML algorithms into sensors and wearable technologies to ensure coaches and athletes can fully utilize these data for injury prevention and further improving sports performance [5,6].

Future research needs to establish evidence and guidelines on using various sensors and wearable technologies in exercise and sports. Marketing and proclamations of commercially available wearable sensors in sports are usually overstated and unsubstantiated [7]. In order to improve the efficacy and effectiveness of using wearable sensors to reduce injury and improve the performance of athletes at various levels and degrees of functioning (disability), more coordinated research efforts among large sports organizations are urgently needed. To date, there is no evidence on the long-term use of wearable sensors in reducing sports injuries [8]. A multidisciplinary team needs to be formed to work with sports organizations for coordinated collection, validation, and interpretation of longitudinal sensor data and athletes’ performance and injury history to establish evidence and guidelines on using sensors and wearable technologies in sports.
